# The diagnostic accuracy of digital PCR, ARMS and NGS for detecting KRAS mutation in cell-free DNA of patients with colorectal cancer: A protocol for systematic review and meta-analysis

**DOI:** 10.1097/MD.0000000000020708

**Published:** 2020-06-26

**Authors:** Peng Ye, Peiling Cai, Jing Xie, Yuanyuan Wei

**Affiliations:** a Department of Anatomy and Histology, College of Medicine, Chengdu University; b Department of Pathology and Clinical Laboratory, Sichuan Provincial Fourth People's Hospital; c Department of Physiology, College of Medicine, Chengdu University, Chengdu, China.

**Keywords:** accuracy, cell-free DNA, colorectal cancer, KRAS

## Abstract

**Introduction:**

Cetuximab and panitumumab have been used clinically to treat metastatic colorectal cancer for more than 15 years. Before the treatment is given, it is required to determine the KRAS mutation status since it would lead to drug resistance. Tumor tissue sample is traditionally used for cancer genotyping. In recent years, liquid biopsy sample has been intensively investigated as a surrogate for tumor tissue sample due to its non-invasiveness and better presentation of tumor heterogeneity. The aim of this study is to systematically summarize the accuracy of KRAS mutation measurement in colorectal cancer using cell-free DNA in liquid biopsy samples, with tumor tissue sample as reference (gold standard).

**Methods and analysis:**

We will search literatures in the following databases: Pubmed, Embase, and Cochrane Library. Systemic review and meta-analysis will be performed to summarize the accuracy of KRAS mutation measurement in colorectal cancer using liquid biopsy sample, and subgroup analysis will be performed on different testing platforms, and on metastatic and non-metastatic colorectal cancer.

**Timeline:**

This study will start on June 1, 2020, and is expected to be finished by November 1, 2020.

**Ethics and dissemination:**

Ethical approval will not be required since the data obtained and analyzed in this study will not be on individual patients. Study results will be disseminated as an official publication in a peer-reviewed journal.

**Registration:** PROSPERO CRD42020176682

## 1 Introduction

Currently, colorectal cancer is still a leading cause of cancer-related death worldwide.^[1]^ Surgery remains mainstay of treatment for colorectal cancer, but for non-resectable tumors, chemotherapy, and targeted therapy are mostly used.^[2]^ An example of the targeted therapy for colorectal cancer is anti-epithelial growth factor receptor (EGFR) therapy, e.g., cetuximab and panitumumab, which have been used for the treatment of metastatic colorectal cancer for more than 15 years.^[3]^ However, those targeted therapies were plaqued by drug resistance. For example, somatic mutations of KRAS gene in tumor can cause resistance to anti-EGFR therapy, which makes it necessary to test KRAS mutation status before the therapy is given.^[4]^

The detection of KRAS mutation in colorectal cancer is mostly performed on tumor tissue sample, but for recurrent or metastatic colorectal cancer patients whose tumor tissue samples are not available, liquid biopsy sample (e.g., plasma, urine, etc.) serves as an alternative.^[5]^ In addition, liquid biopsy is a non-invasive approach in cancer genotyping and also could better indicate tumor heterogeneity.^[6,7]^ Using cell-free DNA extracted from liquid biopsy samples, KRAS mutation status can be determined using several techniques, including digital PCR, amplification refractory mutation system (ARMS), and next generation sequencing (NGS).^[8–11]^

### 1.1 Objectives

The primary objective of this study is to assess the accuracy of detecting KRAS mutation status using cell-free DNA in liquid biopsy samples compared to tissue samples. In addition, we also plan to compare the diagnostic accuracy between different detecting methods, including PCR, ARMS, and NGS. The results could guide the use of liquid biopsy in KRAS mutation detection in colorectal cancer. We have performed a thorough search on Pubmed, Embase, Cochrane Library, and PROSPERO, and did not find any other meta-analysis performed on this topic.

## 2 Methods and analysis

### 2.1 Study registration

This study protocol has been registered on PROSPERO (Registration number: CRD42020176682).

### 2.2 Research question development

Research questions were developed following the PICO framework.^[12]^ Please find details in Table 1.

**Table 1:**
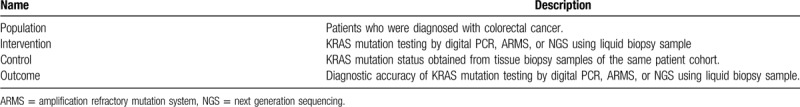
PICO research question development.

### 2.3 Eligibility criteria

Inclusion criteria:

All original studies describing accuracy of KRAS mutation detection in cell-free DNA of patients with colorectal cancer using digital PCR, ARMS, or NGS, or a comparison among those techniques, with tissue samples as reference (gold standard).

Exclusion criteria:

1. not a human study;
2. not describing KRAS mutation;
3. no liquid biopsy samples or tissue samples included;
4. did not use any techniques among digital PCR, ARMS, and NGS;
5. not colorectal cancer;
6. reviews, abstracts, letter to the editor, comments, case reports, or studies with uninterpretable data.

### 2.4 Information source

Pubmed, Embase, and Cochrane Library databases will be searched for eligible studies. No limitation will be applied.

### 2.5 Searching strategy

Searching will be performed using keywords “KRAS”, “digital PCR”, “NGS”, “next generation sequencing”, “ARMS”, “amplification refractory mutation system”, “circulating tumour DNA”, “cell-free DNA”, “liquid biopsy” and “colorectal cancer”. Please see Table 2 for details of searching strategy.

**Table 2:**
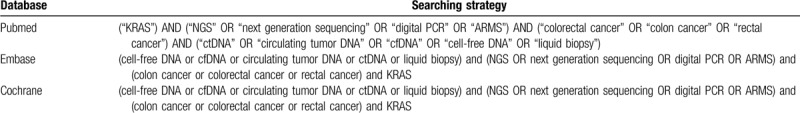
Searching strategy.

### 2.6 Study selection

**Figure 1:**
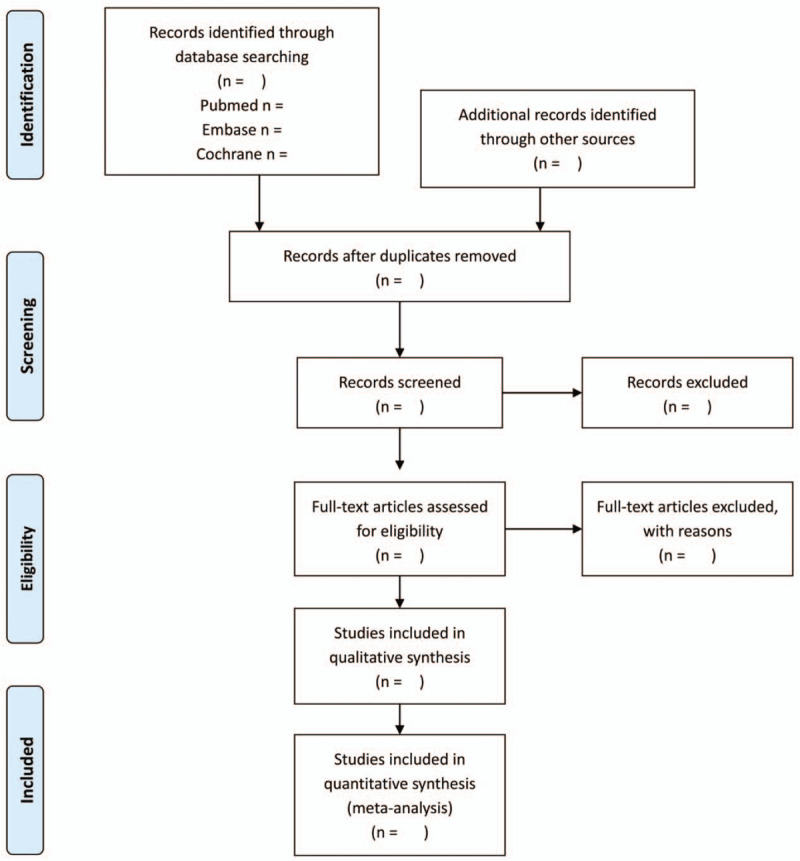
PRISMA 2009 flow diagram.

Eligible studies will be independently searched and screened by 2 researchers (PY and PC). Any disagreement between the 2 researchers will be resolved by a third researcher (YW). Number of excluded studies will be shown in PRISMA flowchart and reasons of exclusion will be provided, as indicated in Figure 1.

### 2.7 Data management

After literature search in online databases, list of the searching results will be recorded by the 2 researchers (PY and PC) and sent to a third researcher (YW). After eligible studies are finalized, full-text of the studies will be downloaded. Data will be extracted using a data extraction table which will be uploaded to Systematic Review Data Repository (SRDR) for record.

### 2.8 Data extraction and collection

Full text of eligible studies will be downloaded and information will be independently extracted by PY and PC using a data extraction table prepared before the information extraction.

### 2.9 Collected data items

After list of eligible studies is finalized, the following information will be collected: author information (name of first author), publication year, characteristics of patients (age, race), testing platform for KRAS mutation in liquid biopsy, and tissue samples (digital PCR, ARMS or NGS), type of liquid biopsy samples (plasma, serum, urine, cerebrospinal fluid, and etc.), sample size, numbers of true positive, false positive, false negative, and true negative.

### 2.10 Study outcomes

The primary study outcome will be diagnostic accuracy of detecting KRAS mutation in cell-free DNA, with KRAS mutation status in the paired tissue biopsy as control. The parameters of diagnostic accuracy evaluated in this meta-analysis will include sensitivity, specificity, positive likelihood ratio (PLR), negative likelihood ratio (NLR), diagnostic odds ratio (DOR), the summary receiver operating characteristic (SROC) curve, and area under curve (AUC). The secondary study outcome will be a comparison between the diagnostic accuracy of digital PCR, ARMS, and NGS in detecting KRAS mutation in cell-free DNA.

### 2.11 Incomplete information and missing data

During the data extraction step, if we find any incomplete or missing information, we will try to contact the author for help. If we fail to obtain those data, the study will be excluded from the final data synthesis.

### 2.12 Risk of bias in individual study

Quality assessment of diagnostic accuracy studies 2 (QUADAS-2) will be used to evaluate each eligible study, which will be independently performed by 2 researchers (PY and PC). Disagreement between the 2 researchers will be resolved by YW.

### 2.13 Statistical analysis and data synthesis

Statistical analysis will be performed using STATA software with MIDAS module and Meta-Disc software version 1.4. Pooled values will be calculated for sensitivity, specificity, PLR, and NLR. DOR will be calculated by PLR divided by NLR. The SROC curve will be generated and AUC will be calculated. Cochrans Q and Thompson *I*^2^ test will be used to examine inter-study heterogeneity. Based on the results of heterogeneity test, fixed-effects model will be used if no significant heterogeneity is detected (*I*^*2*^ ≤ 50%); otherwise, random-effects model will be used (*I*^*2*^ > 50%).

### 2.14 Subgroup analysis

We plan to perform subgroup analysis on the testing platform for KRAS mutation in liquid biopsy (e.g., digital PCR vs ARMS vs NGS), and on metastatic and non-metastatic colorectal cancer, if feasible. In case of significant inter-study heterogeneity, we will try to find possible sources of heterogeneity and perform subgroup analysis if possible.

### 2.15 Publication bias

Begg funnel plot and Egger test will be used to evaluate publication bias.

### 2.16 Confidence in cumulative evidence

Confidence in cumulative evidence will be evaluated following GRADE guideline. Imprecision will be evaluated using sample size and confidence interval of outcomes. Inconsistency will be evaluated by Thompson *I*^2^ test as described in Section 2.13. Indirectness will be evaluated using the PICO information from the eligible studies. Publication bias will be evaluated as described in Section 2.15.

## 3 Discussion

In the era of precision medicine, precise cancer genotyping is very important for the success of targeted therapies. Cancer genotyping in clinical practice is mostly performed using tumor tissue sample (referred as “gold standard”), which includes surgically-resected and biopsy tumor samples. However, the procedure of obtaining tumor tissue sample is invasive and results based on tumor tissue sample could be biased due to tumor heterogeneity.^[13–15]^ Liquid biopsy sample has been intensively investigated for its use as a surrogate of tissue sample in cancer genotyping since its non-invasiveness and better presentation of tumor heterogeneity.^[16–18]^ However, its accuracy and reliability need to be proven. In this study, we propose a protocol for a systematic review and meta-analysis on the accuracy of KRAS mutation detection in colorectal cancer using liquid biopsy sample, with paired tissue sample as control. We hope the results of this study could be used as a reference for the future use of liquid biopsy in KRAS mutation detection in colorectal cancer by clinicians and researchers.

## Author contributions

**Conceptualization:** Peng Ye, Yuanyuan Wei.

**Data curation:** Peng Ye, Peiling Cai.

**Funding acquisition:** Yuanyuan Wei.

**Methodology:** Peng Ye, Jing Xie.

**Project administration:** Yuanyuan Wei.

**Resources:** Peng Ye, Peiling Cai.

**Supervision:** Yuanyuan Wei.

**Writing – original draft:** Peng Ye.

**Writing – review & editing:** Peiling Cai, Jing Xie, Yuanyuan Wei.
